# Looking for the origins of axons

**DOI:** 10.7554/eLife.79839

**Published:** 2022-06-01

**Authors:** Kathleen S Rockland

**Affiliations:** 1 Department of Anatomy and Neurobiology School of Medicine, Boston University Boston United States

**Keywords:** subplate, pyramidal neurons, inhibitory interneurons, axon initial segment, neurofilament, evolution, Human, Mouse, Rat, Rhesus macaque, Other

## Abstract

Pyramidal neurons with axons that exit from dendrites rather than the cell body itself are relatively common in non-primates, but rare in monkeys and humans.

**Related research article** Wahle P, Sobierajski E, Gasterstädt I, Lehmann N, Weber S, Lübke JHR, Engelhardt M, Distler C, Meyer G. 2022. Neocortical pyramidal neurons with axons emerging from dendrites are frequent in non-primates, but rare in monkey and human. *eLife* **11**:e76101. doi: 10.7554/eLife.76101.

Pyramidal neurons are easily recognizable because their soma – the part of the neuron that contains the nucleus – has a characteristic triangular shape (hence their name). On closer examination, however, it becomes clear that the size of the soma can vary, as can the size and shape of the ‘arbor’ formed by the dendrites that carry signals to the soma (DeFelipe and Fariñas, 1992). Moreover, it has been reported that the axons of some pyramidal neurons in the mammalian cortex emerge from dendrites rather than from the base of the soma (Triarhou, 2014; Figure 1).

**Figure 1.**
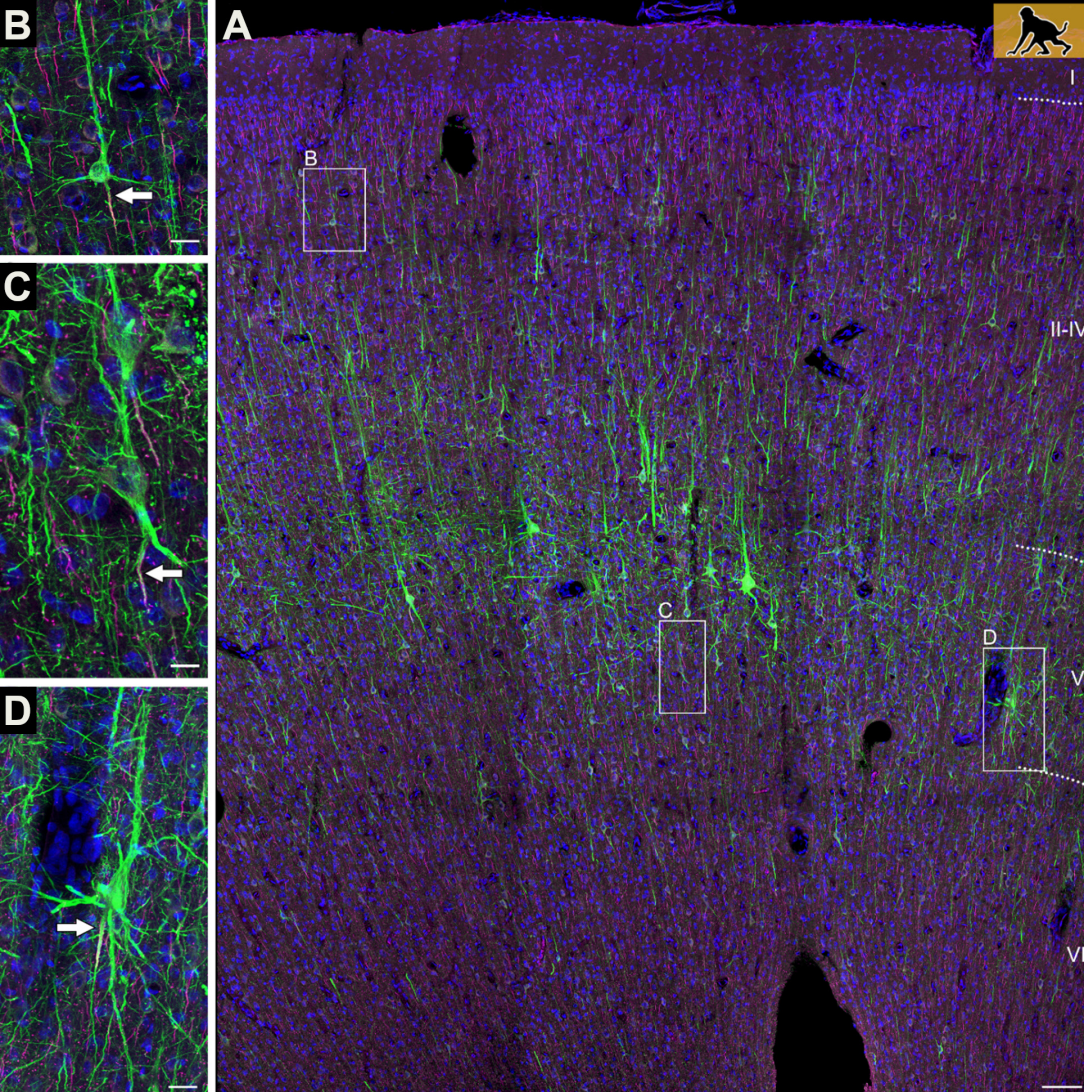
Axons can emerge from different parts of pyramidal neurons. (**A**) The neocortex of an infant macaque monkey that has been stained with dyes that label the dendrites and axons (initial segment only) of pyramidal neurons (shown in green). Branching off the base of the soma are several basal dendrites, and a single apical dendrite protrudes from its apex. Most pyramidal neurons contain an axon (indicated by the white arrow) that exits from the base of the soma (**B**). In some neurons, however, the axon emerges from a dendrite (**C**), or initially co-joins with a dendrite as it exits from the soma (**D**). Scale bars represent 100 micrometers for panel A, and 25 micrometers for the other panels.

Image credit: Adapted from Figure 1 in Wahle et al., 2022

These ‘axon carrying dendrites’ are unusual because the signals dendrites receive are usually processed in the soma before they are sent out via the axon to other neurons (Förster, 2014). These kinds of morphological differences are important because they influence how individual neurons and neuronal groups compute information. Researchers are particularly interested in features that only occur in humans and primates, as these may be associated with cognitive behaviors as well as neurological and psychiatric conditions. Now, in eLife, Petra Wahle from Ruhr University Bochum and co-workers in Germany, Austria and Spain report that the proportion of axon carrying dendrites (AcDs) varies between mammalian species and different areas of the brain (Wahle et al., 2022).

Wahle et al. used a range of histological techniques to compare the morphology and structure of pyramidal neurons in postmortem tissue samples extracted from six cortical areas at different stages of the animals’ development (Figure 2). This revealed that the proportion of pyramidal neurons with an AcD was around 10–20% in non-primate mammals (rat, cat and ferret), but much lower (typically a few percent) in macaque monkeys and humans. Moreover, AcDs were rarely found in the upper layers of the neocortex: these layers are thicker in non-human primates and humans, and are associated with complex behaviors and higher cortical functions.

**Figure 2.**
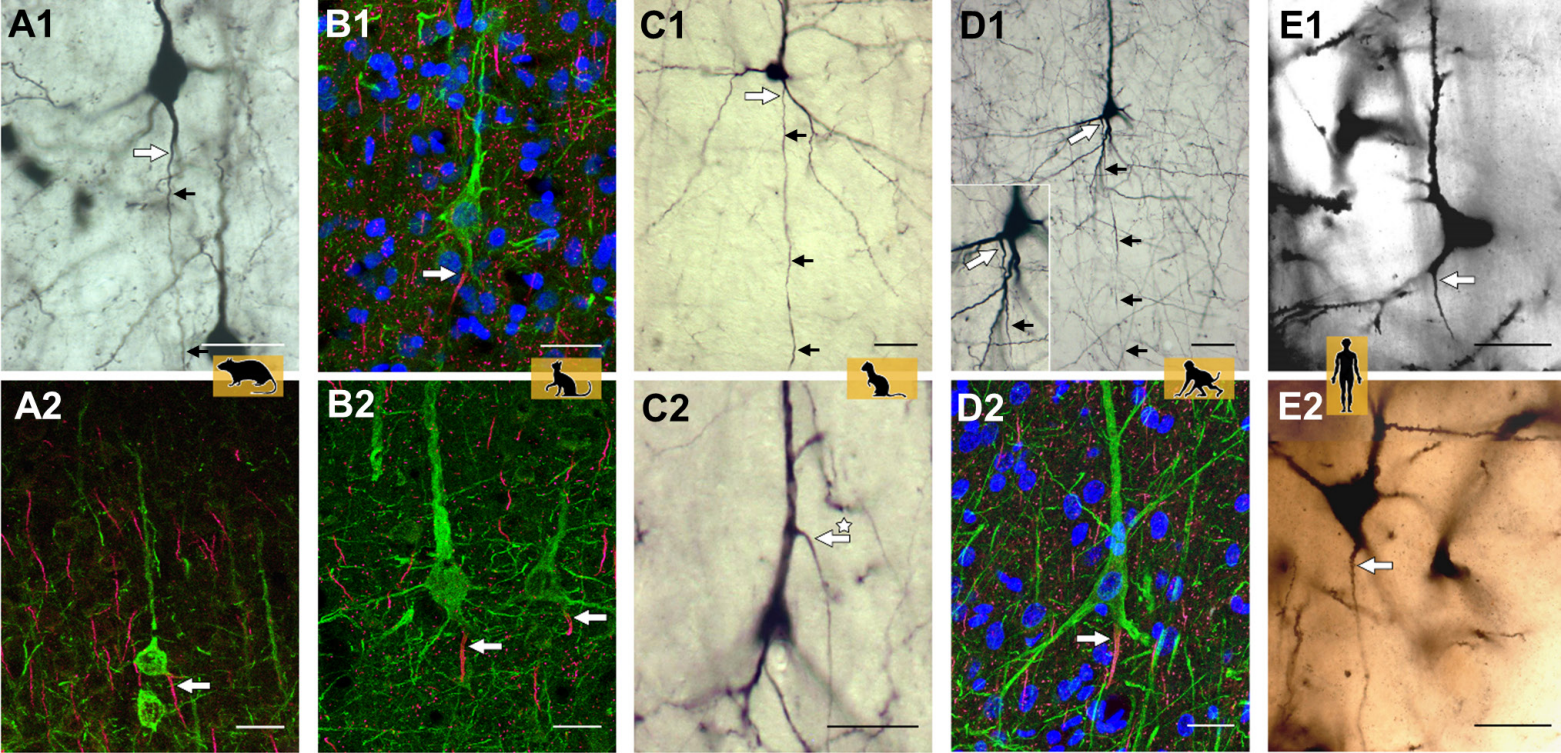
Axon carrying dendrites in different mammalian species. Neurons with axon carrying dendrites (AcDs) in the rat visual cortex (**A1, A2**), the cat visual cortex (**B1, B2**), the ferret visual cortex (**C1, C2**), the macaque premotor cortex (**D1, D2**), and the human auditory cortex (**E1, E2**). Tissue samples were stained using one or two of the following techniques: biocytin (black and white images), immunofluorescence (multi-color images), or the Golgi method (human auditory cortex only). The site where the axons emerge is indicated by a large white arrow, with smaller black arrows highlighting the direction the axon takes through the neocortex. In most pyramidal neurons, the AcD emerges from the base of the soma. It is very rare for the AcD to emerge from the apical dendrite: an example of this is indicated by the asterisk in panel C2. Fewer than ten examples of apical AcDs were identified in the rat, ferret and macaque tissue samples studied, and none were identified in the human tissue samples. Scale bars represent 25 micrometers for all panels.

Image credit: Adapted from Figure 2 in Wahle et al., 2022.

So, what might be the reason for humans and non-human primates having fewer AcDs? It is thought that AcDs enhance the electrical behavior of pyramidal neurons by allowing signals to bypass the soma and flow directly from the dendrite to the axon. This is supported by prior studies showing that AcDs generate stronger and more frequent electrical spikes, and also require a lower threshold to trigger an action potential (Thome et al., 2014; Kole and Brette, 2018). Wahle et al. propose that humans and non-human primates have fewer AcDs because they already have other cellular specializations that can boost the strength of the electrical signal sent through pyramidal neurons.

The careful quantification and systematic survey of pyramidal neurons from different species and brain regions also yielded three other observations. First, Wahle et al. found that inhibitory interneurons do not follow the same trend as pyramidal cells: primates have similar numbers of AcD interneurons as other mammalian species. Second, humans have an unexpectedly high number of AcDs (8.86–13.23%) in the white matter compartment below the cortex, which largely contains bundles of axons intermixed with a population of scattered neurons. Among other features, these neurons are born early in development and have less distinct axon and dendritic regions compared to neurons at the cortical surface (Suárez-Solá et al., 2009). This weakened polarity may be the reason why a higher proportion of neurons in white matter have AcDs.

Third, consistent with previous work on the hippocampus (Benavides-Piccione et al., 2020), Wahle et al. found that humans had more pyramidal neurons with AcDs in this region of the brain than mice – which is the opposite of what happens in other parts of the cortex. Wahle et al. suggest that this could be because the hippocampus of humans and non-human primates requires extra features to carry out its complex memory-related processes. One way to test this could be to expand the histological analysis to other parts of the brain that are also associated with higher order functions, such as the amygdala.

In a broader context, the work by Wahle et al. adds to an already long list of pyramidal neurons that are morphologically distinct (DeFelipe and Fariñas, 1992). This includes inverted pyramidal neurons where apical dendrites (which usually emerge from the top of the soma) orientate down towards the white matter rather than up towards the surface of the brain (Banovac et al., 2021). An anatomical study in rabbits showed that the axons of inverted neurons can exit from multiple places (the base or side of the cell body, or off the downward-facing dendrite), and can project to a variety of cortical structures (Mendizabal-Zubiaga et al., 2007). This mixed population of standard and inverted pyramidal neurons has also been found in the brains of non-primate mammals and the hippocampus of non-human primates, suggesting that they may lead to different axonal outputs.

Another possibility is that differences in morphology may result from mis-steps during development. Notably, inverted pyramidal neurons are abundant in the cortex of mutant reeler mice, which have disorganized cortical layers owing to disruptions in neuronal migration during development (Landrieu and Goffinet, 1981; Förster, 2014).

Organization of the cytoskeleton (the scaffold of proteins that maintains the cell’s structure) may also play a role in these morphological differences, particularly the location of the initial segment of the axon, which varies across vertebrates and invertebrates (Prokop, 2020). Species variations are becoming increasingly easy to investigate due to the advent of super-resolution microscopy (Xu et al., 2013), and AcDs offer an interesting assay for examining ultrastructural specializations in normal and abnormal brains.

The findings of Wahle et al. highlight how the morphology of pyramidal neurons, and potentially other cells in the brain, does not always follow a uniform pattern and can vary between species. The study also offers several new avenues of research. For instance, the mechanisms involved in the formation and function of AcDs remain to be explored. Furthermore, it is still unclear why in the samples studied by Wahle et al. it is more common for dendrites at the base of the pyramidal soma to have AcDs than the apical dendrite (Figure 2C). In addition, other mechanisms – such as gene expression and metabolism – might play a role in this unusual axon location. AcDs could be an effective assay to identify neurons with distinct roles in the brain (Höfflin et al., 2017).

Answering these questions will require expanding the histological approach used by Wahle et al. to other areas of the brain, especially regions outside the cortex, and examining more species and stages of development. In addition, computer simulations could be used to model the consequences of having different proportions of AcDs. This could provide new insights into how and why cognitive behavior differs so much between species, particularly humans and non-human primates.
